# Olfactory Loss in Rhinosinusitis: Mechanisms of Loss and Recovery

**DOI:** 10.3390/ijms25084460

**Published:** 2024-04-18

**Authors:** Agnès Dekeyser, Caroline Huart, Thomas Hummel, Valérie Hox

**Affiliations:** 1 Laboratory of Pneumology, ENT (Airways) and Dermatology (Skin) (LUNS), Institute of Experimental and Clinical Research (IREC), UCLouvain, 1200 Brussels, Belgium; agnes.dekeyser@uclouvain.be (A.D.); caroline.huart@saintluc.uclouvain.be (C.H.); 2 Department of Otorhinolaryngology, Head and Neck Surgery, Cliniques Universitaires Saint-Luc, 1200 Brussels, Belgium; 3 Smell and Taste Clinic, Department of Otorhinolaryngology, University Hospital Carl Gustav Carus, TU Dresden, 01307 Dresden, Germany; thomas.hummel@tu-dresden.de

**Keywords:** olfaction, olfactory dysfunction, chronic rhinosinusitis

## Abstract

Chronic rhinosinusitis (CRS) is a highly prevalent disease and up to 83% of CRS patients suffer from olfactory dysfunction (OD). Because OD is specifically seen in those CRS patients that present with a type 2 eosinophilic inflammation, it is believed that type 2 inflammatory mediators at the level of the olfactory epithelium are involved in the development of this olfactory loss. However, due to the difficulties in obtaining tissue from the olfactory epithelium, little is known about the true mechanisms of inflammatory OD. Thanks to the COVID-19 pandemic, interest in olfaction has been growing rapidly and several studies have been focusing on disease mechanisms of OD in inflammatory conditions. In this paper, we summarize the most recent data exploring the pathophysiological mechanisms underlying OD in CRS. We also review what is known about the potential capacity of olfactory recovery of the currently available treatments in those patients.

## 1. Introduction

“Olfaction” or “the sense of smell” is a chemosensory system that plays a major role in our daily life. Besides its role in detecting environmental hazards, it also has a great influence on our nutritional and social behaviours.

Although olfactory dysfunction (OD) is usually considered a trivial problem, it can severely impact an individual’s quality of life. Patients encounter mood changes, difficulties enjoying and preparing food lead to decreased appetite and weight changes, safety and vulnerability issues arise due to the difficulty to detect spoiled food, gas, or fire-related smoke, insecurities by not detecting their body odor, decreased ability to perform at the workplace especially when olfaction is required, and difficulties in social and sexual interactions [1,2,3]. The interest in smell and the consequences of OD grew dramatically during the SARS-CoV-2 pandemic in 2019, when a substantial part of the population suddenly experienced the disadvantages linked to OD. This has brought OD to the forefront of public awareness, leading to a steep increase in scientific publications on the subject [2].

Olfactory perception may occur through two different pathways: the ortho- and retronasal routes. Orthonasal perception occurs via odorants that are inhaled through the nose and reach the olfactory cleft. Retronasal perception comes from odorants originating from the oral cavity (i.e., food) that reach the olfactory cleft through the oro- and nasopharynx. This retronasal perception plays an important role in flavour perception and is often mistakenly confused with taste [2,4].

**Figure 1:**
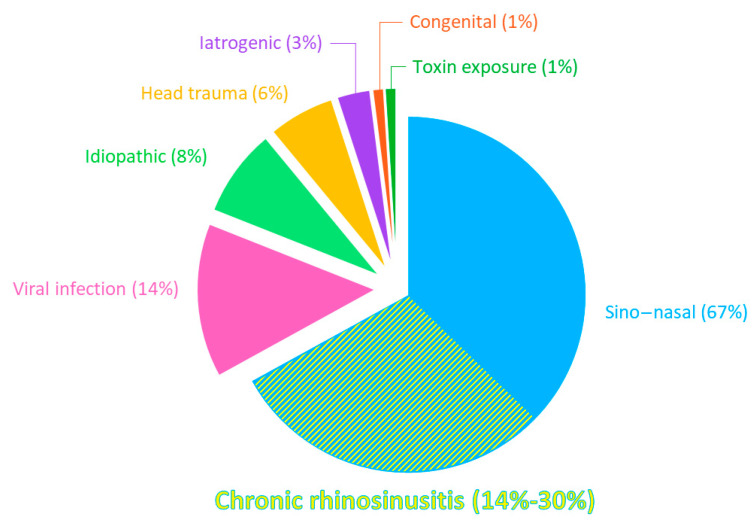
Distribution of olfactory loss aetiologies. Percentages of OD causes in patients treated in different German, Austrian, and Swiss ENT clinics. Adapted from Damm, Schmitl [5].

OD has been described as conductive, sensorineural, or central. Conductive OD results from the blocking of the nasal cavity, thereby preventing odorants from reaching the olfactory epithelium. Sensorineural OD occurs when neurons are unable to transmit the sensory information due to OE damage. Finally, OD is described as central if caused by injury of the central pathways [2]. Apart from aging, the leading cause of OD is sino-nasal diseases, defined by an inflammation of the nose and/or paranasal sinuses, being responsible for 67% of OD in patients presenting to smell centres [5]. Among the sino-nasal diseases, chronic rhinosinusitis (CRS) is the main cause of OD, accounting for 14–30% of cases (Figure 1) [2,5,6]. OD is mostly a well-known hallmark symptom of CRS, with it even being adopted in the definition of CRS [7]. OD can also occur in allergic rhinitis but is less prevalent and less severe than in CRS [7,8].

CRS is an inflammatory disease of the nasal mucosa and paranasal sinuses and is a frequent health problem affecting 5 to 12% of the general population [7]. It is characterised by symptoms of nasal congestion, rhinorrhoea, facial pressure, and smell loss lasting for more than 12 weeks [7]. In addition to the substantial socio-economic burden, due to healthcare costs, patients’ absenteeism at work, or patients’ loss of work productivity, CRS is known to have a serious impact on the quality of life of affected individuals [9].

CRS can be classified into CRS with (CRSwNP) or without (CRSsNP) nasal polyps. Interestingly, in CRSwNP patients, OD is considered as one of the most frustrating aspects of the disease, highlighting the burden of OD [10]. Although most studies only analysed the orthonasal perception, some others found that retronasal perception is also impacted in CRS patients [11,12,13] but less than orthonasal function [11]. Unfortunately, mechanisms of OD in CRS remain largely unknown, which hampers an adequate management of this invalidating phenomenon.

In this review, we aim to summarize the most recent knowledge on pathophysiology of CRS-induced OD. We also review what is known about the smell recovering capacities of the currently available treatments for OD in CRS. By doing so, we want to highlight the knowledge gaps and pinpoint the unmet needs in basic and clinical research.

## 2. Epidemiology

Olfactory dysfunction (OD) is quite common. A recent systematic review estimated it to impact up to 29% of the general adult population [14]. This review used data from studies recruiting patients in the general population, not only focusing on a specific disease or olfactory complaints.

When looking at the different causes of OD, we have to rely on a population of patients presenting themselves in specialized smell and taste clinics. In this population, olfactory loss has been shown to be multifactorial, with causes being sino-nasal diseases (67%), viral infections (14%), head trauma (6%), iatrogenic causes (3%), exposure to toxins (1%), and congenital abnormalities (1%) (Figure 1) [5]. In 8% of the cases, no clear cause could be detected, and those patients are addressed as suffering from idiopathic OD [5]. It is important to mention that due to a previous lack of interest in OD, a substantial number of OD patients never consulted a smell specialist for their problem and are managed by their general practitioner or not managed at all. Therefore, the true real-life numbers of causal factors might be skewed. 

As mentioned above, the major sino-nasal disease that causes OD is CRS, rather than allergic rhinitis. A systematic review showed that 78% of CRS patients were found to suffer from OD [15]. When focusing on the different CRS phenotypes, OD prevalence rose to 94% in CRSwNP patients [15]. Regarding the CRSsNP subgroup, numbers are lacking since studies do not tend to report this phenotype as a distinct subgroup in their analysis [15]. It must be noted that studies investigating OD epidemiology are very heterogeneous, assessing olfaction by means of different, and sometimes non-validated, olfactory tests. Some of these studies are based on self-rated smell tests, which have a poor reliability since it has been shown that the average sensitivity of self-rated OD was not higher than 20% [16].

OD associated with sino-nasal disease shows some specific characteristics compared to other types of OD. CRS-related OD tends to occur gradually, and fluctuates over time [17]. Fluctuation of OD is found to be a factor closely associated to CRS-related OD. Also, OD in CRS rarely improves without treatment and parosmia is not commonly present [18,19,20].

## 3. The Olfactory System in the Healthy Individual

Most of the human nasal mucosa is comprised of the non-olfactory multi-layered respiratory mucosa [21]. The olfactory epithelium (OE) is located in the higher regions of the nasal cavity. It covers the medial part of the superior turbinate, the anterior, middle, and superior part of the middle turbinate, and the posterior region of the septum [22,23]. It presents different cell types: the sensory olfactory neurons (OSNs), the sustentacular cells, the microvillar cells, Bowman’s gland, and the basal cells [21,24]. OSNs are neurons that transmit olfactory information, sustentacular cells are supporting cells with protective functions toward neurons, Bowman’s glands produce the mucus covering the OE, and basal cells form the progenitor compartment of the OE. Microvillar cells have a so far unknown, probably chemosensory, function in the OE [21,24].

When individuals breathe or swallow, odorants are carried to the OE. At the mucosal level, these odorants bind to the olfactory binding proteins (OBPs) in order to cross the mucus layer that covers the OE [25]. Once they reach the OE, they can bind and activate the olfactory receptors (OR) present on the cilia of the OSNs, inducing their depolarisation. The subsequent olfactory signal is sent to the central part of the olfactory system via the olfactory nerves, which are formed by the axons of OSNs. OSNs express the same OR project to the same glomerulus. Of note, odorants can bind to different receptors and receptors recognize different odorants. The perceived odor would result from a pattern of activation [24]. 

Following organization through the olfactory bulb, the information reaches the olfactory cortex (consisting of the anterior olfactory nucleus, olfactory tubercle, piriform cortex, anterior cortical amygdaloid nucleus, periamygdaloid cortex, and lateral entorhinal cortex) and other brain regions such as the orbitofrontal cortex, the insular cortex, the thalamus, the hypothalamus, and hippocampus [21,24]. 

Due to its direct contact with the external environment, the OE is vulnerable to damage caused by toxins, pathogens, or trauma. Those would constantly impact its olfactory function were it not for its ability of neurogenesis [26,27,28,29,30,31,32,33,34]. 

In 1940, it was shown that the OE undergoes a continuous turnover through life and is able to regenerate after injury. This is elicited by two types of multipotent olfactory progenitor cells situated at the basal pole of the epithelium: globose basal cells (GBCs) and horizontal basal cells (HBCs). Several studies demonstrated that both types of basal cells can regenerate both neuronal and non-neuronal cells of the OE [33,35,36,37,38,39,40]. In normal and acute conditions, GBCs are believed to be responsible for the turnover of the OE. HBCs, on the other hand, are believed to start differentiating only in case of severe injury [24,27,35,41,42].

## 4. Disease Mechanisms of Olfactory Loss in Chronic Rhinosinusitis

### 4.1. Conductive Problems

Several decades ago, it was believed that OD in CRS was due to a conduction problem, resulting from the obstruction of the nasal airways by nasal polyps or congestion of the nasal mucosa [43]. Computational modelling and imaging studies have indeed demonstrated that small changes in the volume of specific parts of the nasal cavity, such as the nasal valve region or the upper and inferior meatus, might modify the airflow and the effectiveness of odorants in reaching the olfactory cleft (OC) [44,45]. Some patients even present with a specific anatomical obstruction of the OC (olfactory cleft syndrome), leading to a decreased olfactory sensitivity [46].

However, the conductive hypothesis alone cannot explain all ODs observed in CRS, since we know that a substantial number of patients retain an OD despite surgery to remove polyps or increase the nasal airflow [7].

**Figure 2:**
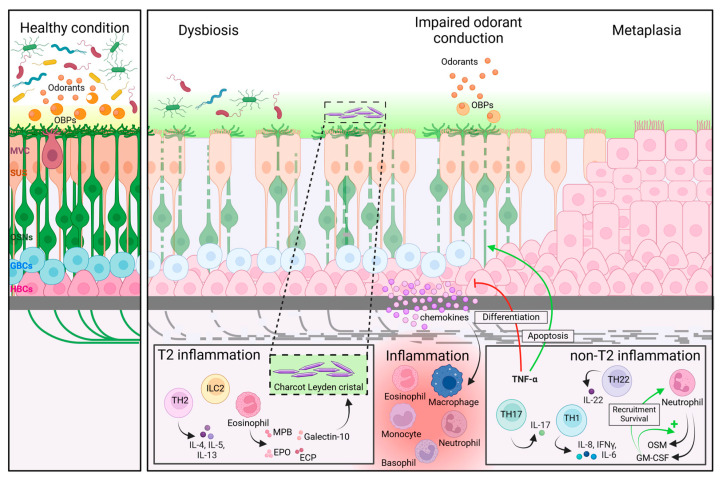
Possible disease mechanisms of OD in CRS. Possible mechanistic pathways that can explain the OD observed in CRS patients, based on available scientific data. OBPs: olfactory binding proteins. MVC: microvillar cells. SUS: sustentacular cells. OSNs: olfactory sensory neurons. GBCs: globose basal cells. HBCs: horizontal basal cells. TH: T helper. ILC2: type 2 innate lymphoid cells. IL: interleukin. IFNγ: interferon-γ. TNF-α: tumour necrosis factor α. MBP: major basic protein. EPO: eosinophil peroxidase. ECP: eosinophil cationic protein. OSM: oncostatin M. GM-CSF: granulocyte-macrophage colony-stimulating factor. Created with BioRender.com.

The problem of odorant conduction might also be at the level of the OC mucus (Figure 2). The mucus layer that covers the OE contains proteins such as OBPs that facilitate the penetration of odorants through the mucus layer, or olfactory metabolizing enzymes that eliminate odorants. These proteins are known to play an important role in transmitting the olfactory signal [47,48]. A study on 30 CRS patients found an altered proteomic profile of the OC mucus in both CRSwNP and CRSsNP patients, showing fewer OBPs and metabolizing enzymes in CRS compared to controls. One can speculate that this alteration of OC mucus proteome in CRS might lead to an inadequate transmission of odorants to the OE, resulting in OD [25].

### 4.2. Local Inflammation

In addition to odorant conduction problems, other studies suggest that inflammation at the level of the sino-nasal mucosa plays a role in the development of OD in CRS (Figure 2) [43,49,50,51]. As mentioned above, CRS is characterized by two phenotypes, CRSwNP and CRSsNP. In the more recent guidelines, this phenotype-based classification has shifted to a categorization based on the sino-nasal inflammatory endotype [7]. Endotypes are defined as either type 2 (T2) or type 1/type3 (non-T2) inflammation, depending on the primary cytokines and effector cells involved. T2 inflammation is characterized by the presence of type 2 innate lymphoid cells (ILC2s) and T helper (Th) 2 cells that produce IL-4, IL-5, and IL-13 and recruit eosinophils to the nasal and sinus mucosa. Non-T2 inflammation is characterized by the secretion of more general inflammatory cytokines such as IL-8, IFNy, and IL-6 but also IL-17 and IL-22, being produced, respectively, by Th1, Th17, or Th22 cells. They typically recruit neutrophils to the nasal and sinus mucosa [52]. It is important to note that mixed endotypes exist and that the association between endotypes and phenotypes varies according to geographical regions. In Western countries, CRSwNP is usually associated with a T2 inflammation, and Western CRSsNP patients present with a 50-50 division among the two endotypes [53,54]. Asian CRSwNP patients, however, present more frequently with a non-T2 endotype, as do patients that present with CRSwNP and a cystic fibrosis background [55,56].

A biopsy study from 2000 has shown that the OE of CRS patients is capable of mounting an inflammatory response involving the influx of inflammatory cells such as lymphocytes, macrophages, and eosinophils [43]. The presence of these inflammatory cells was, in most cases, linked to a defective olfactory function, as measured by the UPSIT smell test. The authors also showed degeneration of the OE that was overlying pockets of these inflammatory cells, suggesting that this chronic inflammation can be toxic to OSNs, leading to cell death and subsequent OD [43]. The toxic features might depend on the type of inflammation present at the level of the OE.

#### 4.2.1. Type-2 Inflammation

Eosinophils are the major effector cells of the T2-inflammation endotype [57]. Eosinophils are known to release proteins that are cytotoxic for epithelia, such as major basic protein (MBP), eosinophil cationic protein (ECP), and eosinophil peroxidase (EPO) (Figure 2) [58,59]. In his biopsy study from 2000, Kern showed the presence of eosinophils in the lamina propria of the OE, as well as extensive inflammatory response around the nerve bundles in a severe CRSwNP patient [43]. A study in 65 CRSwNP patients demonstrated that a positive correlation existed between SST scores and blood eosinophilia [60]. In a biopsy study from 2017, the presence of eosinophils in superior turbinate biopsies of 73 CRS patients was shown to correlate with OD, as measured by SST, even after controlling for nasal polyps’ status [61]. Eosinophils are also known to produce galectin-10, a protein that forms Charcot Leyden crystals, which are a marker of cell death and are mostly seen in patients with severe CRSwNP (Figure 2) [62]. Liu and colleagues found elevated galectin-10 levels in the OC mucus and superior turbinate biopsies of CRS patients presenting with OD when compared to CRS patients without OD, as well as a negative correlation between galectin-10 level and olfactory score, as measured by SST [63]. T2-associated cytokines could also contribute to the destruction of the OE (Figure 2). A clinical study looking at nasal secretions showed a link between nasal IL-5 levels and psychophysical tested smell reduction in CRS patients [64]. A second study on a Chinese population demonstrated that the presence of T2 inflammatory cytokines IL-4 and IL-5 in mucus from the middle meatus of CRSwNP patients positively correlated with the severity of OD measured by SST [65].

These findings from human studies are also supported by murine data. A company-sponsored study demonstrated impaired neurogenesis in a validated mouse model of T2 CRS based on ovalbumin (OVA) and *S. aureus* enterotoxin administration [66]. Although they were unable to prove OD in these mice by using an olfactory encephalogram and behavioural tests, transcriptomic and histology analyses indicated an increase in OSN apoptosis in the eosinophilic-rich OE with decreased numbers of immature olfactory neurons, possibly due to a decreased cell renewal.

Another more recent murine study using a model of OVA/Aspergillus induced T2 CRS was able to show an OD by means of behavioural testing, and confirmed the presence of T2 inflammation at the level of the olfactory bulb with the presence of reduced matures olfactory sensory neurons [67]. 

#### 4.2.2. Non-Type 2 Inflammation

The non-T2 inflammation endotype, which is mostly seen in CRSsNP, is typically characterized by an influx of neutrophils in the sino-nasal mucosa [68]. Neutrophils produce granulocyte-macrophage colony-stimulating factor (GM-CSF) and oncostatin M (OSM). GM-CSF is a chemoattractant for neutrophils; it will induce their production of OSM and increase their survival. Chronic production of OSM was shown to induce barrier dysfunction of nasal epithelial cells (Figure 2) [69].

To our knowledge, no studies have been performed that demonstrate the presence of neutrophils in the OE of (non-T2) CRS patients. This can be explained by the fact that from the few studies that have been performed, most of them focused on T2 CRSwNP patients. Animal studies, however, have shown (for example, in hamsters) that neutrophilic influx of the OE after SARS-CoV-2 infection in hamsters was the driving factor for epithelia disruption [70]. Therefore, it can be speculated that the OE from non-T2 or mixed-type CRS patients might be invaded with neutrophils that contribute to the epithelial destruction and subsequently to OD. 

One study in the Chinese CRS population has investigated the role of non-T2 cytokines in CRS-related OD. They found a negative correlation between the presence of the T1-inflammatory cytokine TNF-α in mucus from the middle meatus of CRSwNP patients and SST results [65]. A 4-month treatment with TNF-α inhibitors was also shown to significantly improve the olfactory function, measured by SST, of 12 patients suffering from another inflammatory disease, inflammatory bowel disease [71].

This finding is also supported by data from animal experiments. A mouse model of inducible olfactory inflammation, through controlled expression of TNF-α in the OE, showed decreased odorant response by electro-olfactogram recordings and loss of OSNs. The histologic changes observed were reversible once the inflammation was not induced. Authors hypothesized that inflammatory cytokines act on neuronal sensitivity through a calcium-mediated pathway or induce recruitment of inflammatory cells leading to damage of OSNs axons by macrophages and apoptosis (Figure 2) [72]. Furthermore, the knocking-out of the TNF-α receptor 1 (TNFR1) or receptor 2 (TNFR2) in the same mouse model prevented neuronal loss [73,74]. OSN apoptosis due to TNF-α simulation was also observed previously in vitro in organotypic cultures developed from rat olfactory mucosa [75].

TNF-α regulates cell proliferation, differentiation, and apoptosis through the NF-κB and JNK pathway and caspase cascade [76,77]. In a more recent study using the same inducible inflammation mouse model, the inflammatory TNF-α effect on OE was shown to be mediated through the NF-κB pathway. During chronic inflammation, HBCs would prevent the regeneration of the olfactory epithelium by shifting from differentiating to dormant state. They also participate actively in the inflammation by producing NF-κB-related chemokines, themselves recruiting inflammatory cells such as macrophages (Figure 2). The authors hypothesized that this mechanism would be a strategy adopted by the cells to maintain their ability to efficiently repair the epithelium once the inflammation is resolved [78]. Inhibition of basal progenitor cell proliferation could be mediated through TNFR2 activation [74].

### 4.3. Other Potential Mechanistic Drivers

In addition to the inflammatory mediators and conduction problems, the microbiome might also play a role in CRS-induced OD. Although it has been shown that the general number of microbes in the nose and sinuses is similar between CRS patients and controls, CRS is characterized by a reduced microbial diversity at the level of the sino-nasal tract, which is defined as dysbiosis (Figure 2) [49]. Dysbiosis has already been shown in OD patients with a significantly decreased diversity in anosmic patients compared to hyposmic and normosmic, but only when focusing on the discrimination score of the SST [79]. Interestingly, in CRS patients, this reduction in microbial diversity has shown to be more pronounced in patients with OD compared to patients without OD, as measured by SST; their composition differs between the two groups [49].

Another phenomenon that could contribute to the CRS-induced OD is olfactory metaplasia (Figure 2). It has been demonstrated that after injury of the OE, some areas are not regenerated, and instead show some squamous or respiratory morphology. This condition is seen in sino-nasal disease when recurrent infection or epithelial damage induces irreversible damage, resulting in permanent OD [80]. In a biopsy study from 2010, both nasal biopsies of CRS patients and healthy controls showed a heterogeneous OE with squamous metaplasia by immunohistochemistry [81]. However, severe squamous metaplasia, determined qualitatively by observation of OE layers and cells shape, was only observed in CRS patients. Results also showed differences in the number of normal OSNs with 24% in CRS patients compared to 45% in healthy controls as well as a more severe erosion of the OE in CRS patients [81]. A more recent study observed OE regeneration after surgical lesion of rat OE [82]. Resections of the olfactory mucosa present on the nasal septum were analysed by immunohistochemistry at 0, 30, and 90 days after surgery. The authors observed regeneration of the OE after 30 days followed by its degeneration into squamous or ciliated epithelia after 90 days with presence of connective tissue [82]. Furthermore, the influx of macrophages in the tissue was higher after 90 days than after 30 days, questioning the role of inflammation in metaplasia [82]. This OE metaplasia was also shown in a rat lesioned OE. Rats were exposed to methylbromide gas for 6 h in order to injure their OE. Results showed increased cellular proliferation after 1 or 2 days followed by a regeneration of a morphologic normal OE after 8 weeks [33]. However, some areas present impaired regeneration of OE and transition to a respiratory epithelium [33].

### 4.4. Central Abnormalities

It needs to be noted that OD in CRS is also associated with an alteration of the central olfactory system since it was demonstrated that the olfactory bulb volume was diminished in CRS patients. The most probable explanation is a bottom-up effect resulting from sensory deprivation and/or injury of the OSNs decrease projections to the olfactory bulb, reducing its volume [83]. A second study investigating volumetry of central olfactory-related brain regions showed no difference in OB volume in CRS patients compared to healthy controls but a reduced volume of grey matter in some regions of the secondary olfactory cortex, such as the orbitofrontal cortex, the right insula, and the left thalamus in CRS patients with severe OD [84]. Processes behind the impairment of the central regions remain unknown but it may contribute especially to long-lasting smell loss in CRS patients.

### 4.5. Trigeminal System

In addition to the olfactory bulb system, the nasal mucosa is also innervated by a second sensory nerve, the trigeminal system. Many odorants also activate the intranasal chemosensory trigeminal system where they produce cooling and other somatic sensations such as tingling, burning, or stinging [85]. In addition to odorants, the trigeminal nerve is also activated by all kinds of chemical, mechanical, and thermal stimuli that are entering the nose [85]. As is seen for the olfactory system, several studies showed a decreased trigeminal sensitivity in CRS patients [86,87,88,89] probably due to an increase in the trigeminal threshold [89]. They showed that the effect of inflammation on the trigeminal system could depend on the duration of the inflammation with acute inducing hypersensibility and chronic hyposensibility [90]. Another explanation might be the acquired olfactory loss that has shown to lead to reduced trigeminal sensitivity [89].

## 5. Current Treatment Options for CRS-Induced OD and Their Impact on Olfactory Recovery

Management of CRS, including olfactory symptoms, is based on either European [7] or American [91] guidelines. The guidelines are generally built around three main pillars of treatment modalities: standard medical therapy, surgery, and biotherapy [2].

### 5.1. Standard Medical Therapy

Due to its general and potent anti-inflammatory characteristics, corticosteroids by different administration routes are considered to be the mainstay therapy of CRS [7]. The most recent position paper on OD recommend the use of systemic (short courses) and/or intranasal (long-term) corticosteroids for managing OD in patients with CRS [2], taking into account the potential and well-known side effects of these drugs [92].

#### 5.1.1. Topical Intranasal Corticosteroids

A large systemic review including 18 randomised controlled trials and more than 2700 CRS patients found moderate quality evidence for the use of intranasal corticosteroids in the improvement of CRS-induced OD [93]. However, the included studies reported very inconsistent results.

This variable effect of topical corticosteroids on OD could be explained by the fact that the narrow OC is situated in the superior apex of the nasal cavities around 7 cm away from the vestibulum, and the resulting difficulty to be reached by topical drugs. This could be worsened by the presence of anatomical abnormalities such as septal deviations. Several studies have investigated this issue [93], showing that nasal spray is mostly deposited in the anterior part of the nasal cavity, while drops can reach further parts of the nasal cavity depending on the head position [93,94,95]. In the Kateiki position, where the patient lies on his/her side with the head tilted and chin turned upwards [93], nasal drops have been shown to reach the OC in 96% of decongested noses and in 75% of the non-decongested noses, and therefore seems to be the preferred choice. High-volume devices that deliver more than 50 mL to the nasal cavities, such as squeeze bottles and irrigation devices, might perform better [93], as shown by two studies where irrigation reached the olfactory cleft in most cases [95,96].

#### 5.1.2. Oral Corticosteroids

Systemic corticosteroids have generally been shown to be more effective than topical corticosteroids in the treatment of CRS-related OD. A systematic review from 2014 found eight randomized control trials including more than 400 CRS patient with OD [97]. Subjective improvement of olfaction after a short course of oral steroids was reported over placebo in seven of them. Two of these studies also included psychophysical smell testing, confirming the beneficial effect of oral steroids over placebo. Five of these studies investigated the combination of a two-week course of oral steroids followed by a course of topical steroids of a variable duration. Considering psychophysical smell testing, they all failed to show a beneficial effect at more than 12 weeks after stopping the oral steroid course.

The only study that showed a beneficial effect of oral steroids in the long-term is a more recent Greek study of 140 CRSwNP patients that was published after the systematic review. They showed that CRSwNP patients treated with 1 week of oral dexamethasone (0.1 mg/kg/d) and oral omeprazole (20 mg/d), followed by 12 weeks of budesonide nasal spray (200 ug/d) and nasal douches, presented a significant improvement of psychophysical tested olfaction compared to CRSwNP patients treated with the budesonide spray and nasal douching only. This difference between both groups was significant after 2 weeks and remained so after 12 and 24 weeks of treatment [98]. According to these results, it seems that the combination of oral and intranasal corticosteroids significantly improves olfaction in CRSwNP patients at least in the short term.

Little is known about the potential regenerative effect of corticosteroids on the OE. In general corticosteroids diffuse across the cell membranes to bind to the glucocorticoid receptor. This will lead to the transcription of anti-inflammatory genes leading to an inhibition of inflammatory cells and mediators [99]. At the level of the respiratory epithelium, they have also been shown to increase the epithelial integrity and barrier effect [100,101]. At the level of the OE, however, no data are available in the human setting. In an in vitro study with murine OE, it has been shown that the application of dexamethasone on neurospheres derived from mouse OE cells showed a reduced neurosphere formation in steroid-treated cells [102]. Authors showed the impact of the steroids on the mTOR pathway, leading to impaired protein synthesis. Although these results should be interpreted with caution because of the ex vivo non-human setting and the difficulties translating local drug concentrations, these results might indicate that corticosteroids could have a negative effect on the regenerative capacities of the OE. More studies are needed to confirm these findings in and ex vivo and to determine whether this is a true phenomenon.

#### 5.1.3. Other Medical Treatments

Apart from steroids, long-term antibiotics are included in the guidelines as a possible treatment option of CRS in case of failure of other medical treatments. To our knowledge, only one study investigated the effect of oral doxycycline on olfactory loss in CRSwNP patients. Patients were treated for 20 days (decreasing from 200 to 100 mg/d), and their olfaction was measured at baseline and after 1, 2, 4, 8, and 12 weeks. Results did not show any effect of treatment on olfaction after 12 weeks compared to baseline and placebo [103]. The MACS study also evaluated the effect of one antibiotic, azithromycin, on olfactory function in CRSwNP and CRSsNP patients and did not find any significant difference either between treatment and placebo after 12 weeks [104].

Another possible treatment is targeting fungi that are thought to exaggerate the inflammatory response in CRS. A study on 116 CRS patients, testing their sense of smell by mean of a visual analogue scale, did not show any significant difference between placebo and treatment with amphotericin B after three months [105].

Finally, the effect of two months of herbal treatment on olfactory loss in patients with sino-nasal disease was also tested as an alternative treatment but no significant difference was seen between treated and control groups [106].

### 5.2. Surgery

For CRS patients remaining symptomatic despite adequate medical treatment, functional endoscopic sinus surgery (FESS) is the treatment of choice. The goal of this surgery is to remove diseased and/or polypoid tissue and to open up the sinus cavities between the orbits and the skull base in order to have better access of topical treatments to the sinus mucosa. Apart from polyp debulking, the region of the OC is generally left untouched. Although Cochrane reviews have been published on the effectiveness of surgery in CRS, olfaction is not readily discussed as an outcome. Despite this, a meta-analysis on studies assessing olfaction after FESS for CRS showed that olfaction improved in CRS patients undergoing FESS according to nearly every measure of subjective and objective olfaction [107]. Differences were seen based on polyp state and the severity of preoperative OD. The surgical benefit seemed to be better in CRSwNP compared to CRSsNP. In addition, anosmic patients seemed to show a better OD improvement compared to hyposmic and normosmic patients [108]. Primary FESS patients showed a stronger improvement in olfactory function compared to patients undergoing revision surgery [109]. The updated position paper on OD currently recommends FESS for OD caused by all types of CRS [2].

Mechanistically, ESS improves the conduction of the odorants towards the OE by removing the obstructive polyps and therefore allowing the odorants to reach the OC. It also facilitates the contact of topical nasal steroids with the OE after surgery. Interestingly, two studies showed that post-operative olfactory improvement in CRS patients after FESS was associated with an increase in bulb volume [110] and a functional and structural plasticity of the central olfactory system at the level of the orbitofrontal cortex, anterior cingulate cortex, insular cortex, and temporal poles with an increased activity but a decreased volume of grey matter [111]. This suggests that surgery may facilitate neurogenesis at the olfactory mucosa with an additional effect on the central olfactory system.

### 5.3. Biotherapy

Monoclonal antibodies are increasingly being used in the treatment of CRS. Because of their pricing and unknown adverse effects in the long-term, they are reserved for patients with severe and uncontrolled disease, despite maximal medical and surgical therapy. Currently, three monoclonal antibodies have been approved by European Medicines Agency and Food and Drug Administration for the treatment of type 2 CRSwNP: omalizumab (anti-IgE), mepolizumab (anti-IL5), and dupilumab (anti-IL4Rα) [112].

Omalizumab blocks the activity of circulating IgE and has shown to significantly improve olfaction of CRSwNP patients in the POLYP1 and POLYP2 studies [113]. Their sense of smell was assessed by The University of Pennsylvania Smell Identification Test (UPSIT) at baseline and after 8 weeks, 16 weeks, and 24 weeks of treatment. UPSIT scores significantly improved after omalizumab treatment until the end of screening compared to placebo and baseline [113]. Omalizumab was also shown to significantly improve subjective olfaction in CRSwNP treated patients compared to placebo-treated patients progressively until week 16 [114].

Dupilumab is an antibody directed towards the IL-4 receptor alpha subunit (IL-4Rα), which is a part of both IL-4 and IL-13 receptors and has therefore a dual inhibitory function on these T2 related cytokines. The SINUS-24 and SINUS-52 studies evaluated the effect of dupilumab on CRSwNP patient’s olfaction. The study showed a significant and pronounced reduction of smell loss in CRSwNP patients compared to baseline and placebo-treated patients as measured by the UPSIT at 24 weeks [115].

Mepolizumab is a third monoclonal antibody that binds and blocks circulating IL-5, the main cytokine for eosinophil recruitment and activation. In the SYNAPSE study, CRSwNP were given mepolizumab or a placebo in combination with standard care, and olfaction was assessed using the UPSIT every 8 weeks [116]. Interestingly, this treatment did not show a significant effect on olfactory function after 52 weeks.

Currently, there are no head-to-head studies available that compare the effects of these monoclonal antibodies on olfactory function. However, two recent meta-analyses, including RCTs and real-world studies, suggest a superior effect of dupilumab over both other biotherapies regarding both subjective and objective smell recovery [117,118].

All three of these antibodies are known to block the T2 inflammatory cascade by targeting a different molecule of the T2 pathway. Why blocking the IL-4Rα seems to be more effective in recovering olfactory function than blocking IL-5 or IgE remains unknown. Preliminary evidence has shown that murine OSNs and immature neurons express the IL-4Rα [119]. These researchers also demonstrated that both IL-4 and IL-13 significantly increased calcium uptake in murine OSNs and that intranasal administration of IL-4, but not IL-13, induced anosmia in mice. These findings suggest a potential beneficial effect of blocking IL-4 actions on OD.

### 5.4. Saline Nasal Irrigation

Guidelines in the management of CRS also include nasal irrigation that might improve nasal mucosa function by mechanical clearing, enhancement of mucociliary function, washing of antigens, biofilms and inflammatory mediators, and hydration [7].

Saline nasal irrigation was shown to improve the olfactory dysfunction of CRS patients, as shown by two studies observing a significant improvement of psychophysical tested smell in CRS patients after 1 month of hypertonic Dead Sea salt irrigation or isotonic saline [120,121].

Nasal irrigation is also recommended after surgery. Results of the effect on OD of irrigation following surgery are disputed. Significant improvement of olfactory function was shown in CRS patients following 1 month of saline solution irrigation using both bottle and nasal spray [122], and after 4 months of hypertonic saline irrigation [123]. A third study obtained the same results after analyzing the effect of 6 weeks of saline irrigation, lactated Ringer’s irrigation, or hypertonic saline [124]. All solutions showed a significant improvement, while lactated Ringer’s irrigation showed a significantly better one. On the contrary, two studies analyzing the effect of 2-month treatment with electrolyzed acid water or saline, or 12 weeks treatment with isotonic Sterimar™ or Sinus Rinse™, did not find any significant effect on the sense of smell of CRS patients [125,126].

### 5.5. Olfactory Training

Another safe and effective treatment option that is now widely used for the management of non-sino-nasal OD is olfactory training (OT). OT is the act of regularly sniffing or exposing oneself to robust aromas with the intention of regaining the sense of smell in case of OD [127].

In patients suffering from post-viral, post-traumatic, or idiopathic OD, 12 weeks of OT improved olfactory function in 30% of patients [128]. Another study took into consideration the same causes of OD, adding 12 patients with sino-nasal diseases. These patients were treated with either OT in combination with topical corticosteroids or OT only for 8 months. Olfaction was assessed by SST at baseline and after 4 and 8 months. While the OT and topical corticosteroids group showed a significant improvement of OD after 8 months, patients that received OT only showed a small and non-significant increase of olfactory function after 4 months that was maintained after 8 months [129]. Differences in results between both studies could result from the inclusion of the sino-nasal diseases in the analysis, for which OT effect appears to be limited.

However, the potential positive effect of OT in CRS-induced OD was shown by another study that looked at CRS (with and without nasal polyps) undergoing FESS. The authors showed a significantly higher improvement in olfactory function 3 months after surgery in those patients that followed OT compared to patients that did not perform OT [130]. Interestingly, pre-operative anosmic patients showed more improvement than hyposmic patients [130].

So far, nothing is known about the mechanistic effect of OT in CRS patients with OD. It is, however, known that exposure to odorants can influence the regenerative capacity of the olfactory system [131,132,133], which forms the base of the concept of OT in non-sino-nasal OD. It can be speculated that the persisting inflammatory environment might hamper the effect of OT in sino-nasal OD patients. More studies that examine this very safe and cheap management option for CRS-related OD are definitely needed, possibly in combination with other treatment modalities such as steroids, surgery, and/or biologicals.

### 5.6. Aspirin Desensitization in Non-Steroidal Anti-Inflammatory Drug (NSAID)-Exacerbated Respiratory Disease (NERD)

A certain proportion of CRSwNP suffer from N-ERD and they generally present as the most severe subgroup, with the highest levels of T2 inflammation. Most of them suffer from concomitant asthma and their respiratory symptoms are typically provoked by the ingestion of aspirin or NSAIDs [134]. In these individuals, aspirin or NSAIDs trigger an abnormal immune response, leading to the overproduction of leukotrienes and subsequent inflammation. This reaction is believed to be mediated by the enzyme cyclooxygenase-1 (COX-1), which is inhibited by aspirin and NSAIDs, leading to a shift in arachidonic acid metabolism toward the production of leukotrienes.

The management of NERD patients with CRSwNP is similar to what has been described above, in conjunction with strict avoidance of any NSAID or cross-reactive drugs.

In addition, it has been shown that this condition could be improved by aspirin desensitization, which has also an impact on the OD experienced by those patients [7]. Aspirin treatment after desensitization with oral aspirin (ATAD) was shown to significantly improve olfactory function in CRS patients after 6 months of treatment in several studies [135,136,137].

## 6. Conclusions

OD is highly prevalent in patients suffering from CRS and especially those presenting with nasal polyps. Despite the regenerative ability of the OE, olfaction remains chronically impaired in most CRSwNP patients. The pathophysiology is not well understood because of a lack of studies, partially due to the difficulties of taking biopsies, but interest in OD seems to be rising. Different mechanisms such as conduction problems and OE inflammation probably lie at the base of this invalidating phenomenon, but other mechanisms such as microbial dysbiosis and epithelial metaplasia might also play a role. 

For now, different treatments for CRS are available that deal, in a variable way, with OD, which remains one of the most frustrating symptoms for these patients. We believe that more studies are needed to gain a better insight into disease mechanisms of CRS-induced OD as well as to recovery mechanisms. This will help us to improve our treatment selection and to find better or more cost-effective treatments for affected patients.

The limitation of this review is that it was not addressed in a systematic way. We searched the PubMed, ScienceDirect, and Scopus databases, using the terms “olfactory dysfunction”, “chronic rhinosinusitis”, “mechanisms”, and “treatments”. Hand searching of reference lists was also performed on included studies. 

## References

[1] Temmel A.F.P., Quint C., Schickinger-Fischer B., Klimek L., Stoller E., Hummel T. (2002). Characteristics of olfactory disorders in relation to major causes of olfactory loss. Arch. Otolaryngol. Head Neck Surg..

[2] Whitcroft K.L., Altundag A., Balungwe P., Boscolo-Rizzo P., Douglas R., Enecilla M.L., Fjaeldstad A.W., Fornazieri M.A., Frasnelli J., Gane S. (2023). Position paper on olfactory dysfunction: 2023. Rhinology.

[3] Patel R.M., Pinto J. (2014). Olfaction: Anatomy, physiology, and disease. Clin. Anat..

[4] Huart C. (2014). Novel Psychophysical and Electrophysiological Tools to Assess Human Olfactory Function and Evaluation of Their Potential for an Early Diagnosis of Alzheimer’s Disease. Doctoral Dissertation.

[5] Damm M., Schmitl L., Müller C.A., Welge-Lüssen A., Hummel T. (2019). Diagnostics and treatment of olfactory dysfunction. Hno.

[6] Rombaux P., Huart C., Levie P., Cingi C., Hummel T. (2016). Olfaction in Chronic Rhinosinusitis. Curr. Allergy Asthma Rep..

[7] Fokkens W.J., Lund V.J., Hopkins C., Hellings P.W., Kern R., Reitsma S., Toppila-Salmi S., Bernal-Sprekelsen M., Mullol J., Alobid I. (2020). European Position Paper on Rhinosinusitis and Nasal Polyps 2020. Rhinology.

[8] Stuck B.A., Hummel T. (2015). Olfaction in allergic rhinitis: A systematic review. J. Allergy Clin. Immunol..

[9] Lourijsen E., Fokkens W., Reitsma S. (2020). Direct and indirect costs of adult patients with chronic rhinosinusitis with nasal polyps. Rhinol. J..

[10] Mullol J., Mariño-Sánchez F., Valls M., Alobid I., Marin C. (2020). The sense of smell in chronic rhinosinusitis. J. Allergy Clin. Immunol..

[11] Landis B.N., Giger R., Ricchetti A., Leuchter I., Hugentobler M., Hummel T., Lacroix J.-S. (2010). Retronasal olfactory function in nasal polyposis. Laryngoscope.

[12] Rombaux P., Weitz H., Mouraux A., Nicolas G., Bertrand B., Duprez T., Hummel T. (2006). Olfactory function assessed with orthonasal and retronasal testing, olfactory bulb volume, and chemosensory event–related potentials. Arch. Otolaryngol. Neck Surg..

[13] Othieno F., Schlosser R.J., Storck K.A., Rowan N.R., Smith T.L., Soler Z.M. (2018). Retronasal olfaction in chronic rhinosinusitis. Laryngoscope.

[14] Desiato V.M., Levy D.A., Byun Y.J., Nguyen S.A., Soler Z.M., Schlosser R.J. (2020). The Prevalence of Olfactory Dysfunction in the General Population: A Systematic Review and Meta-analysis. Am. J. Rhinol. Allergy.

[15] Kohli P., Naik A.N., Harruff E.E., Nguyen S.A., Schlosser R.J., Soler Z.M. (2016). The prevalence of olfactory dysfunction in chronic rhinosinusitis. Laryngoscope.

[16] Shu C.-H., Hummel T., Lee P.-L., Chiu C.-H., Lin S.-H., Yuan B.-C. (2009). The proportion of self-rated olfactory dysfunction does not change across the life span. Am. J. Rhinol. Allergy.

[17] Enriquez K., Lehrer E., Mullol J. (2014). The optimal evaluation and management of patients with a gradual onset of olfactory loss. Curr. Opin. Otolaryngol. Head Neck Surg..

[18] Whitcroft K.L., Cuevas M., Haehner A., Hummel T. (2016). Patterns of olfactory impairment reflect underlying disease etiology. Laryngoscope.

[19] Seiden A. (1997). Olfactory Loss Secondary to Nasal and Sinus Pathology.

[20] Jafek B.W., Moran D.T., Eller P.M., Rowley J.C., Jafek T.B. (1987). Steroid-dependent anosmia. Arch. Otolaryngol. Head Neck Surg..

[21] Smith T.D., Bhatnagar K. (2019). Anatomy of the olfactory system. Handb. Clin. Neurol..

[22] Leopold D.A., Hummel T., Schwob J.E., Hong S.C., Knecht M., Kobal G. (2000). Anterior Distribution of Human Olfactory Epithelium. Laryngoscope.

[23] Fitzek M., Patel P.K., Solomon P.D., Lin B., Hummel T., Schwob J.E., Holbrook E.H. (2022). Integrated age-related immunohistological changes occur in human olfactory epithelium and olfactory bulb. J. Comp. Neurol..

[24] Hadley K., Orlandi R.R., Fong K.J. (2004). Basic anatomy and physiology of olfaction and taste. Otolaryngol. Clin. N. Am..

[25] Soler Z.M., Schlosser R.J., Mulligan J.K., Smith T.L., Mace J.C., Ramakrishan V.R., Norris-Caneda K., Bethard J.R., Ball L.E. (2020). Olfactory cleft mucus proteome in chronic rhinosinusitis: A case-control pilot study. Int. Forum Allergy Rhinol..

[26] Schultz E.W. (1941). Regeneration of Olfactory Cells. Proc. Soc. Exp. Biol. Med..

[27] Schultz E.W. (1960). Repair of the olfactory mucosa with special reference to regeneration of olfactory cells (sensory neurons). Am. J. Pathol..

[28] Graziadei P.P.C., Graziadei G.A.M. (1979). Neurogenesis and neuron regeneration in the olfactory system of mammals. I. Morphological aspects of differentiation and structural organization of the olfactory sensory neurons. J. Neurocytol..

[29] Morrison E.E., Costanzo R.M. (1989). Scanning electron microscopic study of degeneration and regeneration in the olfactory epithelium after axotomy. J. Neurocytol..

[30] Jafek B.W., Eller P.M., Esses B.A., Moran D.T. (1989). Post-traumatic anosmia. Ultrastructural correlates. Arch. Neurol..

[31] Burd G.D. (1993). Morphological study of the effects of intranasal zinc sulfate irrigation on the mouse olfactory epithelium and olfactory bulb. Microsc. Res. Tech..

[32] Harding J.W., Getchell T.V., Margolis F.L. (1978). Denervation of the primary olfactory pathway in mice. V. Long-term effect of intranasal ZnSO4 irrigation on behavior, biochemistry and morphology. Brain Res..

[33] Schwob J.E., Youngentob S.L., Mezza R.C. (1995). Reconstitution of the rat olfactory epithelium after methyl bromide-induced lesion. J. Comp. Neurol..

[34] Durante M.A., Kurtenbach S., Sargi Z.B., Harbour J.W., Choi R., Kurtenbach S., Goss G.M., Matsunami H., Goldstein B.J. (2020). Single-cell analysis of olfactory neurogenesis and differentiation in adult humans. Nat. Neurosci..

[35] Leung C.T., Coulombe P.A., Reed R.R. (2007). Contribution of olfactory neural stem cells to tissue maintenance and regeneration. Nat. Neurosci..

[36] Caggiano M., Kauer J.S., Hunter D.D. (1994). Globose basal cells are neuronal progenitors in the olfactory epithelium: A lineage analysis using a replication-incompetent retrovirus. Neuron.

[37] Levey M.S., Chikaraishi D., Kauer J. (1991). Characterization of potential precursor populations in the mouse olfactory epithelium using immunocytochemistry and autoradiography. J. Neurosci..

[38] Schwob J.E., Huard J.M., Luskin M.B., Youngentob S.L. (1994). Retroviral lineage studies of the rat olfactory epithelium. Chem. Senses.

[39] Chen X., Fang H., Schwob J.E. (2004). Multipotency of purified, transplanted globose basal cells in olfactory epithelium. J. Comp. Neurol..

[40] Holbrook E.H., Szumowski K.E.M., Schwob J.E. (1995). An immunochemical, ultrastructural, and developmental characterization of the horizontal basal cells of rat olfactory epithelium. J. Comp. Neurol..

[41] Schwob J.E., Jang W., Holbrook E.H., Lin B., Herrick D.B., Peterson J.N., Coleman J.H. (2016). Stem and progenitor cells of the mammalian olfactory epithelium: Taking poietic license. J. Comp. Neurol..

[42] Child K.M., Herrick D.B., Schwob J.E., Holbrook E.H., Jang W. (2018). The Neuroregenerative Capacity of Olfactory Stem Cells Is Not Limitless: Implications for Aging. J. Neurosci..

[43] Kern R.C. (2000). Candidate’s Thesis: Chronic sinusitis and anosmia: Pathologic changes in the olfactory mucosa. Laryngoscope.

[44] Damm M., Vent J., Schmidt M., Theissen P., Eckel H.E., Lötsch J., Hummel T. (2002). Intranasal volume and olfactory function. Chem. Senses.

[45] Zhao K., Scherer P.W., Hajiloo S.A., Dalton P. (2004). Effect of anatomy on human nasal air flow and odorant transport patterns: Implications for olfaction. Chem. Senses.

[46] Trotier D., Bensimon J.L., Herman P., Tran Ba Huy P., Eloit C. (2007). Inflammatory obstruction of the olfactory clefts and olfactory loss in humans: A new syndrome?. Chem Senses.

[47] Nagashima A., Touhara K. (2010). Enzymatic conversion of odorants in nasal mucus affects olfactory glomerular activation patterns and odor perception. J. Neurosci..

[48] Robert-Hazotte A., Faure P., Neiers F., Potin C., Artur Y., Coureaud G., Heydel J.M. (2019). Nasal mucus glutathione transferase activity and impact on olfactory perception and neonatal behavior. Sci. Rep..

[49] Han X., He X., Zhan X., Yao L., Sun Z., Gao X., Wang S., Wang Z. (2023). Disturbed microbiota-metabolites-immune interaction network is associated with olfactory dysfunction in patients with chronic rhinosinusitis. Front. Immunol..

[50] Soler Z.M., Yoo F., Schlosser R.J., Mulligan J., Ramakrishnan V.R., Beswick D.M., Alt J.A., Mattos J.L., Payne S.C., Storck K.A. (2019). Correlation of mucus inflammatory proteins and olfaction in chronic rhinosinusitis. Int. Forum Allergy Rhinol..

[51] Stevens W.W., Peters A.T., Tan B.K., Klingler A.I., Poposki J.A., Hulse K.E., Grammer L.C., Welch K.C., Smith S.S., Conley D.B. (2019). Associations Between Inflammatory Endotypes and Clinical Presentations in Chronic Rhinosinusitis. J. Allergy Clin. Immunol. Pract..

[52] Macchi A., Giorli A., Cantone E., Pipolo G.C., Arnone F., Barbone U., Bertazzoni G., Bianchini C., Ciofalo A., Cipolla F. (2023). Sense of smell in chronic rhinosinusitis: A multicentric study on 811 patients. Front. Allergy.

[53] Wang X., Zhang N., Bo M., Holtappels G., Zheng M., Lou H., Wang H., Zhang L., Bachert C. (2016). Diversity of T H cytokine profiles in patients with chronic rhinosinusitis: A multicenter study in Europe, Asia, and Oceania. J. Allergy Clin. Immunol..

[54] Delemarre T., Holtappels G., De Ruyck N., Zhang N., Nauwynck H., Bachert C., Gevaert E. (2020). Type 2 inflammation in chronic rhinosinusitis without nasal polyps: Another relevant endotype. J. Allergy Clin. Immunol..

[55] Van Zele T., Claeys S., Gevaert P., Van Maele G., Holtappels G., Van Cauwenberge P., Bachert C. (2006). Differentiation of chronic sinus diseases by measurement of inflammatory mediators. Allergy.

[56] Zhang N., Van Zele T., Perez-Novo C., Van Bruaene N., Holtappels G., DeRuyck N., Van Cauwenberge P., Bachert C. (2008). Different types of T-effector cells orchestrate mucosal inflammation in chronic sinus disease. J. Allergy Clin. Immunol..

[57] Delemarre T., Bochner B.S., Simon H.-U., Bachert C. (2021). Rethinking neutrophils and eosinophils in chronic rhinosinusitis. J. Allergy Clin. Immunol..

[58] Harlin S., Ansel D., Lane S., Myers J., Kephart G., Gleich G. (1988). A clinical and pathologic study of chronic sinusitis: The role of the eosinophil. J. Allergy Clin. Immunol..

[59] Frigas E., Motojima S., Gleich G.J. (1991). The eosinophilic injury to the mucosa of the airways in the pathogenesis of bronchial asthma. Eur. Respir. J. Suppl..

[60] Hox V., Bobic S., Callebaux I., Jorissen M., Hellings P. (2010). Nasal obstruction and smell impairment in nasal polyp disease: Correlation between objective and subjective parameters. Rhinol. J..

[61] Lavin J., Min J., Lidder A.K., Huang J.H., Kato A., Lam K., Meen E., Chmiel J.S., Norton J., Suh L. (2017). Superior turbinate eosinophilia correlates with olfactory deficit in chronic rhinosinusitis patients. Laryngoscope.

[62] Persson E.K., Verstraete K., Heyndrickx I., Gevaert E., Aegerter H., Percier J.-M., Deswarte K., Verschueren K.H.G., Dansercoer A., Gras D. (2019). Protein crystallization promotes type 2 immunity and is reversible by antibody treatment. Science.

[63] Liu Z., Hong J., Huang X., Wu D. (2022). Olfactory cleft mucus galectin-10 predicts olfactory loss in chronic rhinosinusitis. Ann. Allergy, Asthma Immunol..

[64] Schlosser R.J., Mulligan J.K., Hyer J.M., Karnezis T.T., Gudis D.A., Soler Z.M. (2016). Mucous Cytokine Levels in Chronic Rhinosinusitis–Associated Olfactory Loss. JAMA Otolaryngol. Neck Surg..

[65] Han X., Wu D., Sun Z., Sun H., Lv Q., Zhang L., Wei Y. (2020). Type 1/type 2 inflammatory cytokines correlate with olfactory function in patients with chronic rhinosinusitis. Am. J. Otolaryngol..

[66] Rouyar A., Classe M., Gorski R., Bock M.D., Le-Guern J., Roche S., Fourgous V., Remaury A., Paul P., Ponsolles C. (2019). Type 2/Th2-driven inflammation impairs olfactory sensory neurogenesis in mouse chronic rhinosinusitis model. Allergy.

[67] Huang W.-H., Hung Y.-W., Hung W., Lan M.-Y., Yeh C.-F. (2024). Murine model of eosinophilic chronic rhinosinusitis with nasal polyposis inducing neuroinflammation and olfactory dysfunction. J. Allergy Clin. Immunol..

[68] Cho S.H., Kim D.W., Gevaert P. (2016). Chronic Rhinosinusitis without Nasal Polyps. J. Allergy Clin. Immunol. Pract..

[69] Pothoven K.L., Norton J.E., Suh L.A., Carter R.G., Harris K.E., Biyasheva A., Welch K., Shintani-Smith S., Conley D.B., Liu M.C. (2016). Neutrophils are a major source of the epithelial barrier disrupting cytokine oncostatin M in patients with mucosal airways disease. J. Allergy Clin. Immunol..

[70] Bourgon C., Albin A.S., Ando-Grard O., Da Costa B., Domain R., Korkmaz B., Klonjkowski B., Le Poder S., Meunier N. (2022). Neutrophils play a major role in the destruction of the olfactory epithelium during SARS-CoV-2 infection in hamsters. Cell Mol. Life Sci..

[71] Han X., Ordouie A.-A., Schmelz R., Hummel T. (2022). Chemosensory Functions in Patients with Inflammatory Bowel Disease and Their Association with Clinical Disease Activity. Nutrients.

[72] Lane A.P., Turner J., May L., Reed R. (2010). A genetic model of chronic rhinosinusitis-associated olfactory inflammation reveals reversible functional impairment and dramatic neuroepithelial reorganization. J. Neurosci..

[73] Garcia D.S., Chen M., Smith A.K., Lazarini P.R., Lane A.P. (2016). Role of the type I tumor necrosis factor receptor in inflammation-associated olfactory dysfunction. Int. Forum Allergy Rhinol..

[74] Pozharskaya T., Liang J., Lane A.P. (2013). Regulation of inflammation-associated olfactory neuronal death and regeneration by the type II tumor necrosis factor receptor. Int. Forum Allergy Rhinol..

[75] Suzuki Y., Farbman A. (2000). Tumor necrosis factor-alpha-induced apoptosis in olfactory epithelium in vitro: Possible roles of caspase 1 (ICE), caspase 2 (ICH-1), and caspase 3 (CPP32). Exp. Neurol..

[76] Holbrook J., Lara-Reyna S., Jarosz-Griffiths H., McDermott M.F. (2019). Tumour necrosis factor signalling in health and disease. F1000Research.

[77] Victores A.J., Chen M., Smith A., Lane A.P. (2017). Olfactory loss in chronic rhinosinusitis is associated with neuronal activation of c-Jun N-terminal kinase. Int. Forum Allergy Rhinol..

[78] Chen M., Reed R.R., Lane A.P. (2019). Chronic Inflammation Directs an Olfactory Stem Cell Functional Switch from Neuroregeneration to Immune Defense. Cell Stem Cell.

[79] Biswas K., Mackenzie B.W., Ballauf C., Draf J., Douglas R.G., Hummel T. (2020). Loss of bacterial diversity in the sinuses is associated with lower smell discrimination scores. Sci. Rep..

[80] Jafek B., Murrow B., Michaels R., Restrepo D., Linschoten M. (2002). Biopsies of human olfactory epithelium. Chem. Senses.

[81] Yee K.K., Pribitkin E.A., Cowart B.J., Vainius A.A., Klock C.T., Rosen D., Feng P., McLean J., Hahn C.-G., Rawson N.E. (2010). Neuropathology of the olfactory mucosa in chronic rhinosinusitis. Am. J. Rhinol. Allergy.

[82] Mori E., Ueha R., Kondo K., Funada S., Shimmura H., Kanemoto K., Tanaka H., Nishijima H., Otori N., Yamasoba T. (2021). Squamous and Respiratory Metaplasia After Olfactory Mucosal Resection. Front. Neurosci..

[83] Rombaux P., Potier H., Bertrand B., Duprez T., Hummel T. (2008). Olfactory bulb volume in patients with sinonasal disease. Am. J. Rhinol..

[84] Han P., Whitcroft K.L., Fischer J., Gerber J., Cuevas M., Andrews P., Hummel T. (2017). Olfactory brain gray matter volume reduction in patients with chronic rhinosinusitis. Int. Forum Allergy Rhinol..

[85] Hummel T., Frasnelli J. (2019). The intranasal trigeminal system. Handbook of Clinical Neurology.

[86] Saliba J., Fnais N., Tomaszewski M., Carriere J.S., Frenkiel S., Frasnelli J., Tewfik M.A. (2016). The role of trigeminal function in the sensation of nasal obstruction in chronic rhinosinusitis. Laryngoscope.

[87] Migneault-Bouchard C., Lagueux K., Hsieh J.W., Cyr M., Landis B.N., Frasnelli J. (2024). Trigeminal cold receptors and airflow perception are altered in chronic rhinosinusitis. Rhinology.

[88] Burghardt G.K.L., Cuevas M., Sekine R., Hummel T. (2022). Trigeminal Sensitivity in Patients With Allergic Rhinitis and Chronic Rhinosinusitis. Laryngoscope.

[89] Poletti S., Cuevas M., Weile S., Hummel T. (2017). Trigeminal sensitivity in chronic rhinosinusitis: Topographical differences and the effect of surgery. Rhinol. J..

[90] Benoliel R., Biron A., Quek S.Y.P., Nahlieli O., Eliav E. (2006). Trigeminal neurosensory changes following acute and chronic paranasal sinusitis. Quintessence Int..

[91] Orlandi R.R., Kingdom T.T., Smith T.L., Bleier B., DeConde A., Luong A.U., Poetker D.M., Soler Z., Welch K.C., Wise S.K. (2020). International consensus statement on allergy and rhinology: Rhinosinusitis 2021. Int. Forum Allergy Rhinol..

[92] Hox V., Lourijsen E., Jordens A., Aasbjerg K., Agache I., Alobid I., Bachert C., Boussery K., Campo P., Fokkens W. (2020). Correction to: Benefits and harm of systemic steroids for short- and long-term use in rhinitis and rhinosinusitis: An EAACI position paper. Clin. Transl. Allergy.

[93] Espehana A., Lee L., Garden E.M., Klyvyte G., Gokani S., Jegatheeswaran L., Wong J.J., Philpott C. (2023). Delivery of Topical Drugs to the Olfactory Cleft. J. Clin. Med..

[94] Hardy J.G., Lee S.W., Wilson C.G. (1985). Intranasal drug delivery by spray and drops. J. Pharm. Pharmacol..

[95] Scheibe M., Bethge C., Witt M., Hummel T. (2008). Intranasal Administration of Drugs. Arch. Otolaryngol. Neck Surg..

[96] Lam K., Tan B.K., Lavin J.M., Meen E., Conley D.B. (2013). Comparison of nasal sprays and irrigations in the delivery of topical agents to the olfactory mucosa. Laryngoscope.

[97] Banglawala S.M., Oyer S.L., Lohia S., Psaltis A.J., Soler Z.M., Schlosser R.J. (2014). Olfactory outcomes in chronic rhinosinusitis with nasal polyposis after medical treatments: A systematic review and meta-analysis. Int. Forum Allergy Rhinol..

[98] Papadakis C., Chimona T., Chaidas K., Ladias A., Zisoglou M., Proimos E. (2021). Effect of oral steroids on olfactory function in chronic rhinosinusitis with nasal polyps. Eur. Ann. Otorhinolaryngol. Head Neck Dis..

[99] Barnes P.J. (2010). Mechanisms and resistance in glucocorticoid control of inflammation. J. Steroid Biochem. Mol. Biol..

[100] Rimmer C., Hetelekides S., Eliseeva S.I., Georas S.N., Veazey J.M. (2021). Budesonide promotes airway epithelial barrier integrity following double-stranded RNA challenge. PLoS ONE.

[101] Sekiyama A., Gon Y., Terakado M., Takeshita I., Kozu Y., Matsumoto K., Hashimoto S. (2012). Glucocorticoids enhance airway epithelial barrier integrity. Int. Immunopharmacol..

[102] Glezer I., Chang S.Y. (2018). The balance between efficient anti-inflammatory treatment and neuronal regeneration in the olfactory epithelium. Neural Regen. Res..

[103] Van Zele T., Gevaert P., Holtappels G., Beule A., Wormald P.J., Mayr S., Hens G., Hellings P., Ebbens F.A., Fokkens W. (2010). Oral steroids and doxycycline: Two different approaches to treat nasal polyps. J. Allergy Clin. Immunol..

[104] Videler W.J., Badia L., Harvey R.J., Gane S., Georgalas C., Van Der Meulen F.W., Menger D.J., Lehtonen M.T., Toppila-Salmi S.K., Vento S.I. (2011). Lack of efficacy of long-term, low-dose azithromycin in chronic rhinosinusitis: A randomized controlled trial. Allergy.

[105] Ebbens F.A., Scadding G.K., Badia L., Hellings P.W., Jorissen M., Mullol J., Cardesin A., Bachert C., Vanzele T., Dijkgraaf M.G. (2006). Amphotericin B nasal lavages: Not a solution for patients with chronic rhinosinusitis. J. Allergy Clin. Immunol..

[106] Reden J., El-Hifnawi D., Zahnert T., Hummel T. (2011). The effect of a herbal combination of primrose, gentian root, vervain, elder flowers, and sorrel on olfactory function in patients with a sinonasal olfactory dysfunction. Rhinol. J..

[107] Kohli P., Naik A.N., Farhood Z., Ong A.A., Nguyen S.A., Soler Z.M., Schlosser R.J. (2016). Olfactory Outcomes after Endoscopic Sinus Surgery for Chronic Rhinosinusitis: A Meta-analysis. Otolaryngol. Head Neck Surg..

[108] Vandenhende-Szymanski C., Hochet B., Chevalier D., Mortuaire G. (2015). Olfactory cleft opacity and CT score are predictive factors of smell recovery after surgery in nasal polyposis. Rhinology.

[109] Haxel B.R. (2019). Recovery of olfaction after sinus surgery for chronic rhinosinusitis: A review. Laryngoscope.

[110] Gudziol V., Buschhüter D., Abolmaali N., Gerber J., Rombaux P., Hummel T. (2009). Increasing olfactory bulb volume due to treatment of chronic rhinosinusitis—A longitudinal study. Brain.

[111] Whitcroft K.L., Noltus J., Andrews P., Hummel T. (2021). Sinonasal surgery alters brain structure and function: Neuroanatomical correlates of olfactory dysfunction. J. Neurosci. Res..

[112] Fokkens W.J., Viskens A.-S., Backer V., Conti D., De Corso E., Gevaert P., Scadding G.K., Wagemann M., Bernal-Sprekelsen M., Chaker A. (2023). EPOS/EUFOREA update on indication and evaluation of Biologics in Chronic Rhinosinusitis with Nasal Polyps 2023. Rhinol. J..

[113] Gevaert P., Omachi T.A., Corren J., Mullol J., Han J., Lee S.E., Kaufman D., Ligueros-Saylan M., Howard M., Zhu R. (2020). Efficacy and safety of omalizumab in nasal polyposis: 2 randomized phase 3 trials. J. Allergy Clin. Immunol..

[114] Gevaert P., Calus L., Van Zele T., Blomme K., De Ruyck N., Bauters W., Hellings P., Brusselle G., De Bacquer D., van Cauwenberge P. (2012). Omalizumab is effective in allergic and nonallergic patients with nasal polyps and asthma. J. Allergy Clin. Immunol..

[115] Bachert C., Han J.K., Desrosiers M., Hellings P.W., Amin N., Lee S.E., Mullol J., Greos L.S., Bosso J.V., Laidlaw T.M. (2019). Efficacy and safety of dupilumab in patients with severe chronic rhinosinusitis with nasal polyps (LIBERTY NP SINUS-24 and LIBERTY NP SINUS-52): Results from two multicentre, randomised, double-blind, placebo-controlled, parallel-group phase 3 trials. Lancet.

[116] Han J.K., Bachert C., Fokkens W., Desrosiers M., Wagenmann M., Lee S.E., Smith S.G., Martin N., Mayer B., Yancey S.W. (2021). Mepolizumab for chronic rhinosinusitis with nasal polyps (SYNAPSE): A randomised, double-blind, placebo-controlled, phase 3 trial. Lancet Respir. Med..

[117] Oykhman P., Paramo F.A., Bousquet J., Kennedy D.W., Brignardello-Petersen R., Chu D.K. (2022). Comparative efficacy and safety of monoclonal antibodies and aspirin desensitization for chronic rhinosinusitis with nasal polyposis: A systematic review and network meta-analysis. J. Allergy Clin. Immunol..

[118] Barroso B., Valverde-Monge M., Betancor D., Gómez-López A., Villalobos-Vildas C., González-Cano B., Sastre J. (2023). Improvement in Smell Using Monoclonal Antibodies Among Patients with Chronic Rhinosinusitis with Nasal Polyps: A Systematic Review. J. Investig. Allergol. Clin. Immunol..

[119] Hara Y., Jha M.K., Mattoo H., Nash S., Khan A., Orengo J., Hicks A. (2023). Interleukin 4 directly activates olfactory neurons and induces loss of smell in mice. J. Allergy Clin. Immunol..

[120] Friedman M., Hamilton C., Samuelson C.G., Maley A., Wilson M.N., Venkatesan T., Joseph N.J. (2012). Dead Sea salt irrigations vs saline irrigations with nasal steroids for symptomatic treatment of chronic rhinosinusitis: A randomized, prospective double-blind study. Int. Forum Allergy Rhinol..

[121] Savietto E., Marioni G., Maculan P., Pettorelli A., Scarpa B., Simoni E., Astolfi L., Marchese-Ragona R., Ottaviano G. (2020). Effectiveness of micronized nasal irrigations with hyaluronic acid/isotonic saline solution in non-polipoid chronic rhinosinusitis: A prospective, randomized, double-blind, controlled study. Am. J. Otolaryngol..

[122] Macdonald K.I., Wright E.D., Sowerby L.J., Rotenberg B.W., Chin C.J., Rudmik L., Sommer D.D., Nayan S., DesRosiers M., Tewfik M.A. (2015). Squeeze bottle versus saline spray after endoscopic sinus surgery for chronic rhinosinusitis: A pilot multicentre trial. Am. J. Rhinol. Allergy.

[123] Farag A.A., Deal A.M., McKinney K.A., Thorp B.D., Senior B.A., Ebert C.S., Zanation A.M. (2012). Single-blind randomized controlled trial of surfactant vs hypertonic saline irrigation following endoscopic endonasal surgery. Int. Forum Allergy Rhinol..

[124] Low T.-H., Woods C.M., Ullah S., Carney A.S. (2014). A double-blind randomized controlled trial of normal saline, lactated ringer’s, and hypertonic saline nasal irrigation solution after endoscopic sinus surgery. Am. J. Rhinol. Allergy.

[125] Jiang R.-S., Liang K.-L., Wu S.-H., Su M.-C., Chen W.-K., Lu F.-J. (2014). Electrolyzed acid water nasal irrigation after functional endoscopic sinus surgery. Am. J. Rhinol. Allergy.

[126] Salib R., Talpallikar S., Uppal S., Nair S. (2013). A prospective randomised single-blinded clinical trial comparing the efficacy and tolerability of the nasal douching products Sterimar™ and Sinus Rinse™ following functional endoscopic sinus surgery. Clin. Otolaryngol..

[127] Pieniak M., Oleszkiewicz A., Avaro V., Calegari F., Hummel T. (2022). Olfactory training—Thirteen years of research reviewed. Neurosci. Biobehav. Rev..

[128] Hummel T., Rissom K., Reden J., Hähner A., Weidenbecher M., Hüttenbrink K. (2009). Effects of olfactory training in patients with olfactory loss. Laryngoscope.

[129] Fleiner F., Lau L., Göktas Ö. (2012). Active olfactory training for the treatment of smelling disorders. Ear. Nose Throat J..

[130] Park J.Y., Choi B.Y., Kim H., Jung T., Kim J.K. (2022). Olfactory training assists in olfactory recovery after sinonasal surgery. Laryngoscope Investig. Otolaryngol..

[131] Youngentob S.L., Kent P.F. (1995). Enhancement of odorant-induced mucosal activity patterns in rats trained on an odorant identification task. Brain Res..

[132] Wang L., Chen L., Jacob T. (2003). Evidence for peripheral plasticity in human odour response. J. Physiol..

[133] Kim B.Y., Park J., Kim E., Kim B. (2020). Olfactory Ensheathing Cells Mediate Neuroplastic Mechanisms after Olfactory Training in Mouse Model. Am. J. Rhinol. Allergy.

[134] Kowalski M.L., Agache I., Bavbek S., Bakirtas A., Blanca M., Bochenek G., Bonini M., Heffler E., Klimek L., Laidlaw T.M. (2019). Diagnosis and management of NSAID-Exacerbated Respiratory Disease (N-ERD)-a EAACI position paper. Allergy.

[135] Mortazavi N., Esmaeilzadeh H., Abbasinazari M., Babaie D., Alyasin S., Nabavizadeh H., Esmailzadeh E. (2017). Clinical and Immunological Efficacy of Aspirin Desensitization in Nasal Polyp Patients with Aspirin-Exacerbated Respiratory Disease. Ranian J. Pharm. Res. IJPR.

[136] Esmaeilzadeh H., Nabavi M., Aryan Z., Arshi S., Bemanian M.H., Fallahpour M., Mortazavi N. (2015). Aspirin desensitization for patients with aspirin-exacerbated respiratory disease: A randomized double-blind placebo-controlled trial. Clin. Immunol..

[137] Świerczyńska-Krępa M., Sanak M., Bochenek G., Stręk P., Ćmiel A., Gielicz A., Plutecka H., Szczeklik A., Niżankowska-Mogilnicka E. (2014). Aspirin desensitization in patients with aspirin-induced and aspirin-tolerant asthma: A double-blind study. J. Allergy Clin. Immunol..

